# P2RY13+ dendritic cells correlate with enhanced antigen presentation and lymphocyte activation in lung adenocarcinoma

**DOI:** 10.3389/fmed.2025.1708670

**Published:** 2026-01-12

**Authors:** Cong Lan, Jian Zhong, Siyao Che

**Affiliations:** 1 Department of Thoracic Surgery, Affiliated Gaozhou People’s Hospital, Guangdong Medical University, Guangdong, China; 2 Affiliated Gaozhou People’s Hospital, Guangdong Medical University, Guangdong, China

**Keywords:** antigen presentation, dendritic cell, lung adenocarcinoma, P2RY13, T cell

## Abstract

**Background:**

Although P2RY13 has been implicated in immune regulation and prognosis in lung adenocarcinoma (LUAD), its specific cellular expression and functional mechanisms within the tumor microenvironment (TME) remain poorly understood.

**Methods:**

We integrated transcriptomic and clinical data from TCGA and GEO (GSE68465 and GSE31210), performed differential gene expression and functional enrichment analyses, and employed multiple algorithms including CIBERSORT, MCP-counter, quanTIseq, TIMER, and ESTIMATE to evaluate immune infiltration. Single-cell RNA sequencing data (GSE131907) was analyzed using Seurat to identify cell-type-specific expression, while CellChat was used to infer intercellular communication. Pathway activities were assessed with eight scoring methods (AUCell, UCell, AddModuleScore, GSVA, JASMINE, singscore, ssGSEA, viper) using MSigDB gene sets.

**Results:**

Our results demonstrated that P2RY13 was significantly downregulated in LUAD and predicted poor prognosis independently. It correlated positively with immune infiltration, particularly T cell markers. Single-cell analysis revealed specific enrichment in dendritic cells (DCs), with P2RY13(+) DCs more prevalent in normal tissues and exhibiting enhanced activity in antigen presentation and T cell activation.

**Conclusion:**

P2RY13 is an independent prognostic biomarker in LUAD, linked to an immunologically active TME. Its specific expression in dendritic cells enhances antigen presentation and T cell activation, underscoring its role in promoting anti-tumor immunity.

## 1 Introduction

Lung cancer is one of the most prevalent and lethal malignancies worldwide. Histologically, it is classified into non-small cell lung cancer (NSCLC), accounting for about 85% of cases, and small cell lung cancer (SCLC) (1). Among NSCLC subtypes, lung adenocarcinoma (LUAD) is the most common and is notably frequent among non-smokers and younger patients (2). According to recent global estimates, lung cancer leads all cancers in both incidence and mortality, with approximately 2.5 million new cases and 1.8 million deaths annually (3). Despite developments in targeted and immuno-therapies, the prognosis for LUAD remains unfavorable, with a 5-year survival rate below 20%, primarily due to late diagnosis, therapeutic resistance, and tumor heterogeneity (4, 5). Therefore, further investigation into the molecular mechanisms of LUAD and the discovery of novel biomarkers and therapeutic targets are critically needed.

P2RY13, a member of the P2Y purinergic receptor family, is a G protein-coupled receptor implicated in cholesterol transport, bone remodeling, and neuroprotection (6–8). P2RY13 has been shown to exacerbate inflammation in ulcerative colitis (9) and drive carcinogenesis in clear cell renal cell carcinoma through immune mechanisms (10). In addition, P2RY13 has been identified as a prognostic biomarker in LUAD, correlating with improved clinical outcomes and modulating the tumor microenvironment (TME) (11). However, the specific cell types primarily expressing P2RY13 within TME and its precise mechanistic role in modulating immune cell functions remain poorly understood. Further investigation is needed to elucidate how P2RY13 influences the immune landscape in LUAD, particularly through multi-omics approaches to explore its functional impact on TME.

## 2 Materials and methods

### 2.1 Data acquisition

Transcriptomic data and corresponding clinical information for LUAD were obtained from The Cancer Genome Atlas (TCGA) database, comprising 500 tumor samples and 59 adjacent normal tissues. Independent validation dataset GSE68465 and GSE31210 were downloaded from the Gene Expression Omnibus (GEO) database. Protein expression patterns of P2RY13 in LUAD and normal lung tissues were retrieved from the Human Protein Atlas (HPA) database.

### 2.2 Differential expression genes analysis

Patients from the TCGA-LUAD cohort were stratified based on P2RY13 expression levels. The top 200 and bottom 200 samples were defined as the high- and low-expression groups, respectively. Differential expression analysis was performed using DESeq2, Limma, and EdgeR packages in R. Genes with |log₂ fold change| >1.5 and adjusted *p*-value <0.05 were considered significantly differentially expressed. Overlapping differential expression genes (DEGs) identified by all three tools were retained for subsequent analyses.

### 2.3 Functional enrichment analysis

Gene Ontology (GO) and Kyoto Encyclopedia of Genes and Genomes (KEGG) pathway enrichment analyses were conducted on the overlapping DEGs using the “clusterProfiler” package. Gene Set Enrichment Analysis (GSEA) was performed to further identify signaling pathways associated with P2RY13 expression.

### 2.4 Immune infiltration analysis

The association between P2RY13 expression and immune cell infiltration was evaluated using four computational algorithms: CIBERSORT, MCP-counter, quanTIseq, and TIMER. The ESTIMATE algorithm was applied to calculate immune microenvironment scores. Correlation analyses between P2RY13 expression and immune marker genes were performed using Pearson correlation. We calculated the TCellSI scores for different cell states of P2RY13 high and low expression groups from bulk-seq data, comparing the two groups of T cells in the different activation states (12).

### 2.5 Single-cell RNA sequencing data processing

The single-cell RNA-seq dataset GSE131907 was processed and analyzed using Seurat (v5.0). The Seurat package was chosen for its widespread adoption, comprehensive documentation, and well-established workflows, which ensure the reproducibility and comparability of our findings. Quality control, normalization, dimensionality reduction, and clustering were performed following standard workflows. Cell types were annotated based on canonical marker genes. Myeloid subclusters were further re-clustered and annotated into dendritic cells (DCs), macrophages (Mac), monocytes, and undetermined cells.

### 2.6 Analysis of intercellular communication and pathway activity

CellChat was used to infer and compare intercellular communication networks between P2RY13(+) and P2RY13(−) DCs. Differential interaction strengths were evaluated based on ligand-receptor expression patterns. Pathway activity in P2RY13(+) and P2RY13(−) DCs was assessed using eight gene signature scoring methods (AUCell, UCell, AddModuleScore, GSVA, JASMINE, singscore, ssGSEA, viper). This multi-method approach was employed to mitigate potential biases inherent to any single algorithm and to bolster the robustness of the inferred functional differences. Gene sets were obtained from the MSigDB database.

### 2.7 Survival and statistical analysis

Survival analysis was performed using the Kaplan–Meier method and compared with the log-rank test. Multivariate Cox regression models were applied to evaluate the prognostic value of P2RY13 expression. A nomogram was constructed based on independent prognostic factors identified in the TCGA cohort. Calibration curves were plotted to assess the predictive accuracy of the nomogram in both TCGA and GEO cohorts. All statistical analyses were conducted using R software (version 4.1.0).

## 3 Results

### 3.1 Downregulated P2RY13 correlated with worse survival in LUAD

**Figure 1:**
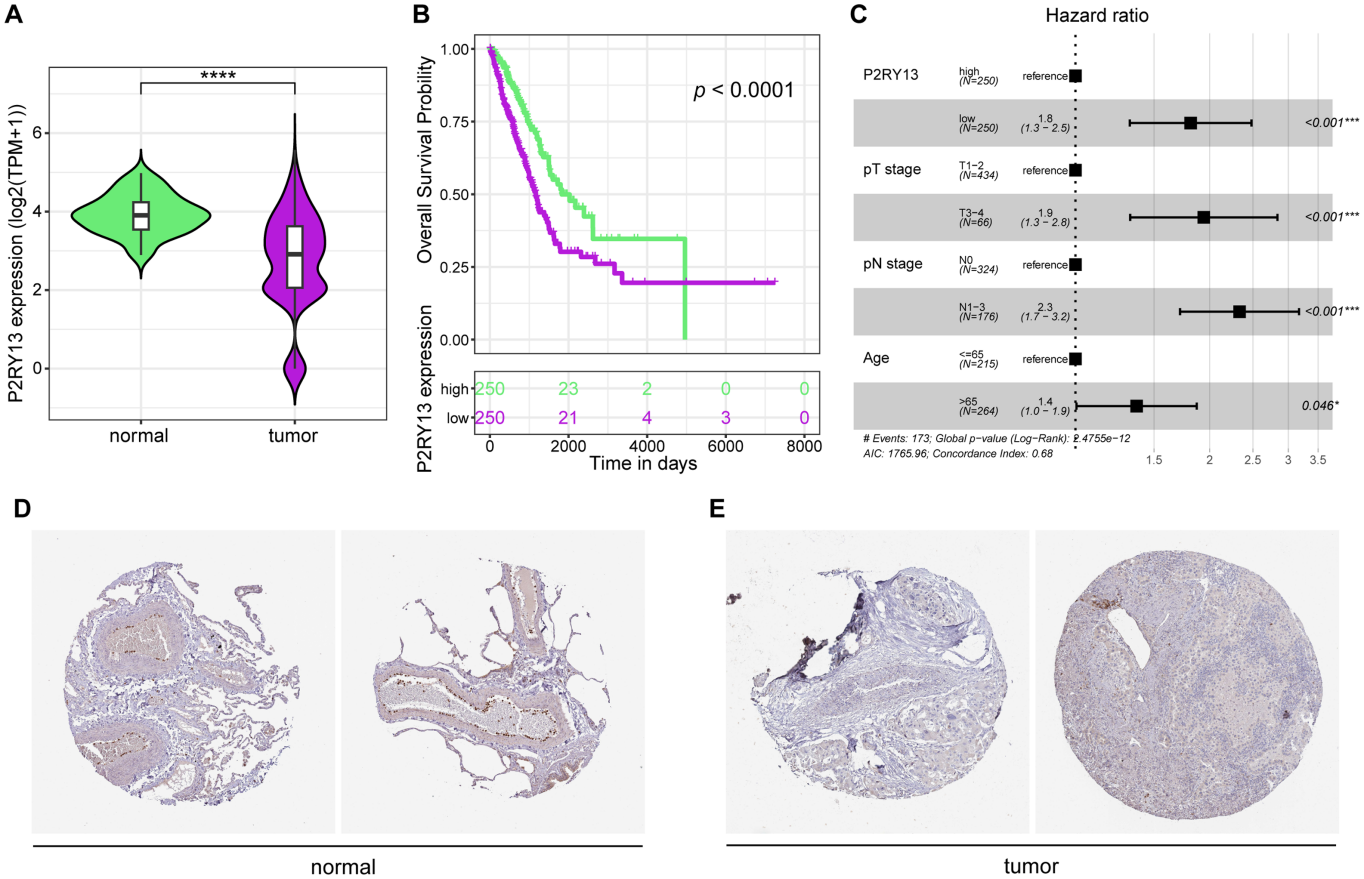
P2RY13 was downregulated in LUAD tumor tissues and correlates with poorer survival. **(A)** P2RY13 expression was significantly reduced in tumor tissues compared to normal tissues in the TCGA-LUAD cohort. **(B)** Patients with low P2RY13 expression showed worse overall survival than those with high expression in the TCGA-LUAD cohort. **(C)** Multivariate Cox regression analysis identified P2RY13 as an independent prognostic factor. **(D,E)** Immunohistochemical analysis from the HPA database confirmed higher P2RY13 expression in normal lung tissues compared to LUAD tissues. ^*^p < 0.05, ^***^p < 0.001, and ^****^p < 0.0001.

In order to explore P2RY13 expression in LUAD, bulk tissue transcriptome data and corresponding survival data were downloaded from TCGA dataset (tumor samples = 500, normal samples = 59). P2RY13 was significantly downregulated in tumor samples (Figure 1A). Low expression of P2RY13 was associated with worse survival (Figure 1B), and P2RY13 expression was identified as an independent factor for survival in LUAD (Figure 1C). As confirmed in IHC analysis of P2RY13 in LUAD patients obtained from HPA dataset (Figures 1D,E), P2RY13 was primarily expressed in the interstitial tissue of normal lung, but is notably absent in LUAD tumor tissues. We hypothesize that P2RY13 may function within the interstitial compartment to mediate the infiltration or modulate the function of stromal cells.

### 3.2 P2RY13 expression was an independent prognostic factor in LUAD

**Figure 2:**
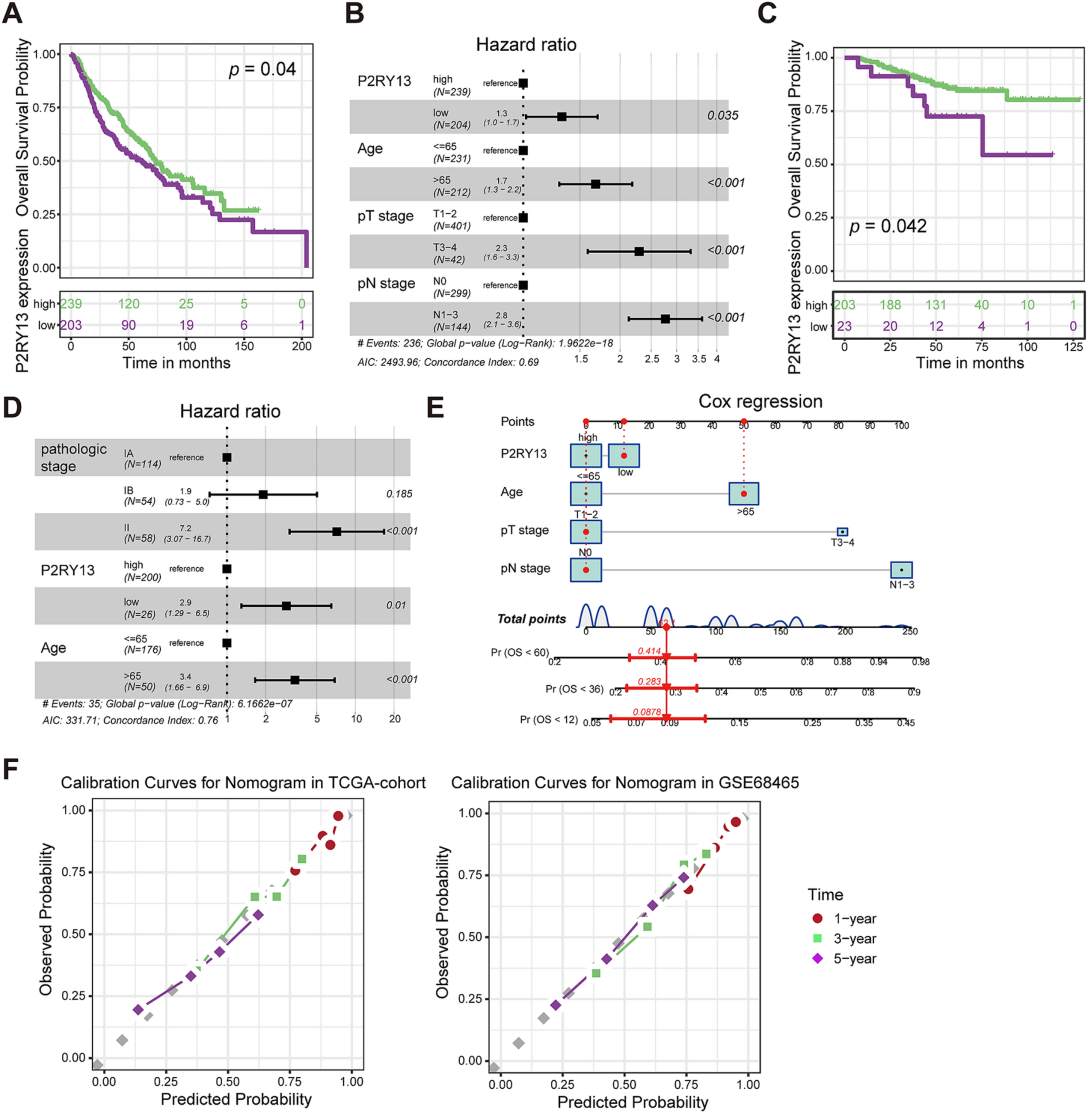
Validation of P2RY13 as an independent prognostic factor in LUAD. **(A)** Kaplan–Meier analysis in GSE68465 validation cohort showed that patients with low P2RY13 expression had significantly poorer survival. **(B)** Multivariate Cox regression analysis confirmed P2RY13 as an independent prognostic factor in validation cohort GSE68465. **(C)** Kaplan–Meier analysis in GSE31210 validation cohort showed that patients with low P2RY13 expression had significantly poorer survival. **(D)** Multivariate Cox regression analysis confirmed P2RY13 as an independent prognostic factor in GSE31210 validation cohort. **(E)** A nomogram predictive model was constructed based on the TCGA-LUAD training cohort. **(F)** Calibration curve for the nomogram in the training cohort (TCGA-LUAD) and the GSE68465 validation cohort.

We further downloaded the transcriptomic and prognostic data from the GSE68465 and GSE31210 datasets to validate the prognostic value of P2RY13 in LUAD patients. As shown in Figures 2A–D, patients with lower P2RY13 expression exhibited worse prognosis, and consistent with the TCGA dataset, P2RY13 served as an independent prognostic predictor. Therefore, we constructed a nomogram (Figure 2E) based on patient age, pT stage, pN stage, and P2RY13 expression in the TCGA cohort. The calibration curves also demonstrated high consistency between predicted and observed outcomes in both the training cohort (TCGA-LUAD, Figure 2F) and the validation cohort (GSE68465, Figure 2F), indicating that the nomogram model incorporating P2RY13 expression can effectively and accurately predict patient prognosis.

### 3.3 Functional enrichment analysis and tumor microenvironment analysis

**Figure 3:**
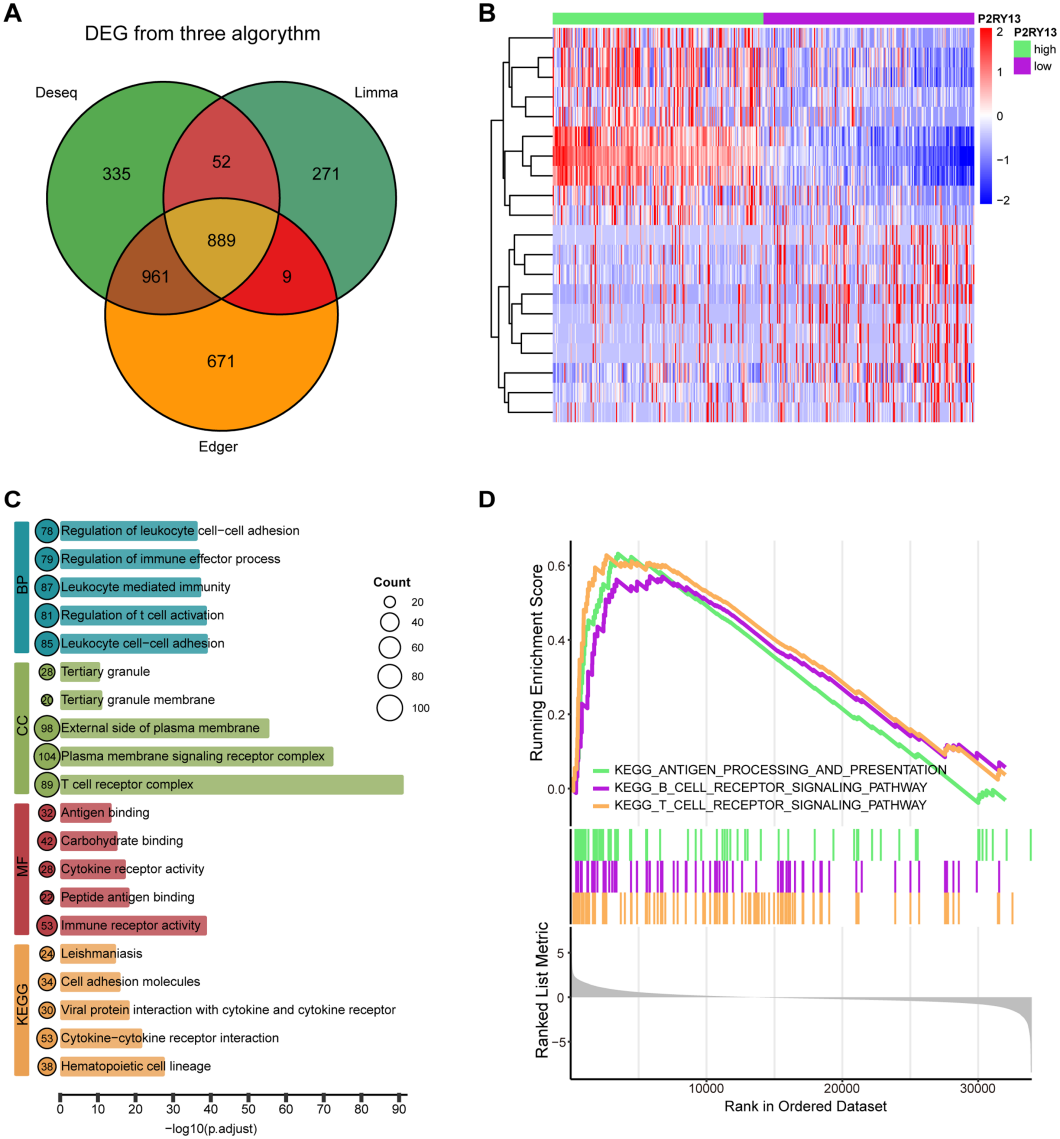
Functional enrichment analysis of DEGs associated with P2RY13 expression. **(A)** Venn diagram of DEGs identified by limma, DESeq2, and EdgeR, with 889 common DEGs. **(B)** Heatmap displaying the top 10 up-regulated and 10 down-regulated genes in patients with the highest (*n* = 200) versus the lowest (*n* = 200) P2RY13 expression. **(C)** Gene Ontology (GO) and Kyoto Encyclopedia of Genes and Genomes (KEGG) enrichment analysis of the 889 common DEGs. **(D)** Gene Set Enrichment Analysis (GSEA) of the 889 DEGs.

To further investigate the role of P2RY13 in LUAD, we stratified 500 tumor samples from the TCGA-LUAD cohort by P2RY13 expression levels and compared those patients with the highest (*n* = 200) and lowest (*n* = 200) P2RY13 expression. DEGs analysis was conducted utilizing the Deseq2, Limma, and EdgeR algorithms. The high-P2RY13 group exhibited 1,150 up- and 1,087 down-regulated genes by Deseq2; 1,006 up- and 215 down-regulated by Limma; and 1,243 up- and 1,287 down-regulated by EdgeR. A consensus set of 889 DEGs common to all three methods was established (Figure 3A). The top 10 up- and down-regulated DEGs are displayed in Figure 3B. GO and KEGG enrichment analyses of the overlapping DEGs revealed associations between P2RY13 expression and immune regulatory processes, including antigen processing, antigen presentation and immune cell activation (Figure 3C). Furthermore, GSEA further confirmed these findings, demonstrating a strong association between high P2RY13 expression and activation of antigen processing and presentation, as well as T cell and B cell receptor signaling pathways (Figure 3D). Taken together, high P2RY13 expression was strongly associated with immune activation, particularly in antigen processing and presentation and T/B cell receptor signaling pathways in LUAD.

**Figure 4:**
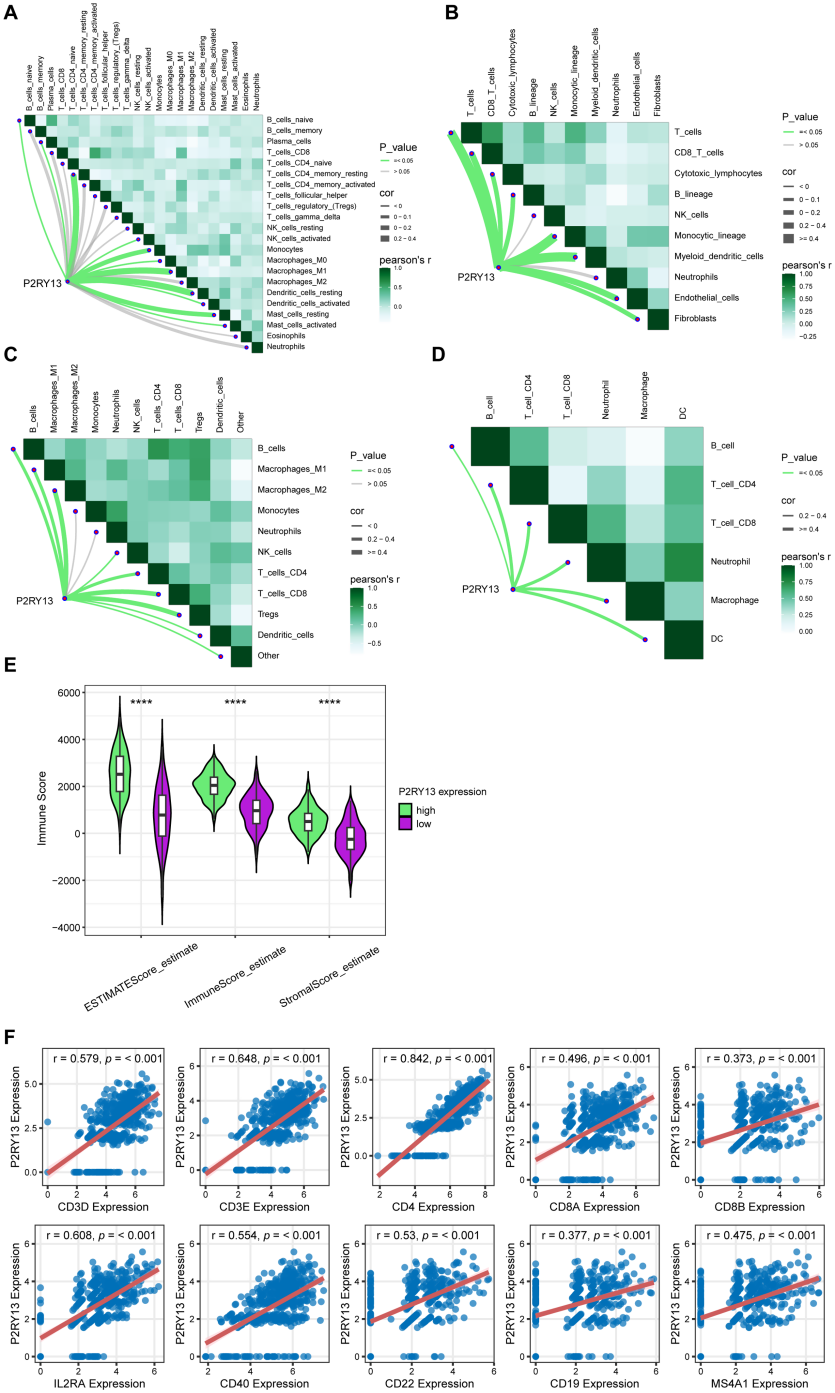
P2RY13 expression is associated with lymphocyte infiltration in LUAD. **(A–D)** Correlation between P2RY13 expression and immune cell infiltration levels, as assessed by four algorithms: **(A)** CIBERSORT, **(B)** MCP-counter, **(C)** quanTIseq, and **(D)** TIMER. **(E)** The ESTIMATE algorithm revealed higher immune microenvironment scores and increased abundance of immune cells in patients with high P2RY13 expression. **(F)** P2RY13 expression was positively correlated with markers of T cells and B cells. ^****^p < 0.0001.

These findings suggest a potential correlation between P2RY13 and the immune microenvironment. We employed multiple computational algorithms (including CIBERSORT, MCP-counter, quanTIseq, and TIMER) to analyze the association between P2RY13 expression and immune cell infiltration within the TCGA-LUAD dataset. The results revealed a significant positive correlation between P2RY13 expression and the level of immune cell infiltration (Figures 4A–D). Furthermore, elevated immune microenvironment scores and a greater abundance of immune cells were observed in patients with high P2RY13 expression by Estimate algorithm (Figure 4E). Consistent with these findings, P2RY13 expression was also positively correlated with CD4 and CD8 expression (Figure 4F), implicating elevated T cell infiltration as a potential underlying mechanism for the anti-tumor role of P2RY13 expression in LUAD microenvironment.

### 3.4 P2RY13 expression was enriched in DCs in LUAD TME

To further delineate the expression and functional role of P2RY13 within the TME, we analyzed the single-cell RNA-seq dataset (GSE131907), focusing specifically on primary LUAD tumors, normal lung tissues, and normal lymph nodes, tissues that most relevant for identifying P2RY13 expression across malignant and normal immunological contexts.

**Figure 5:**
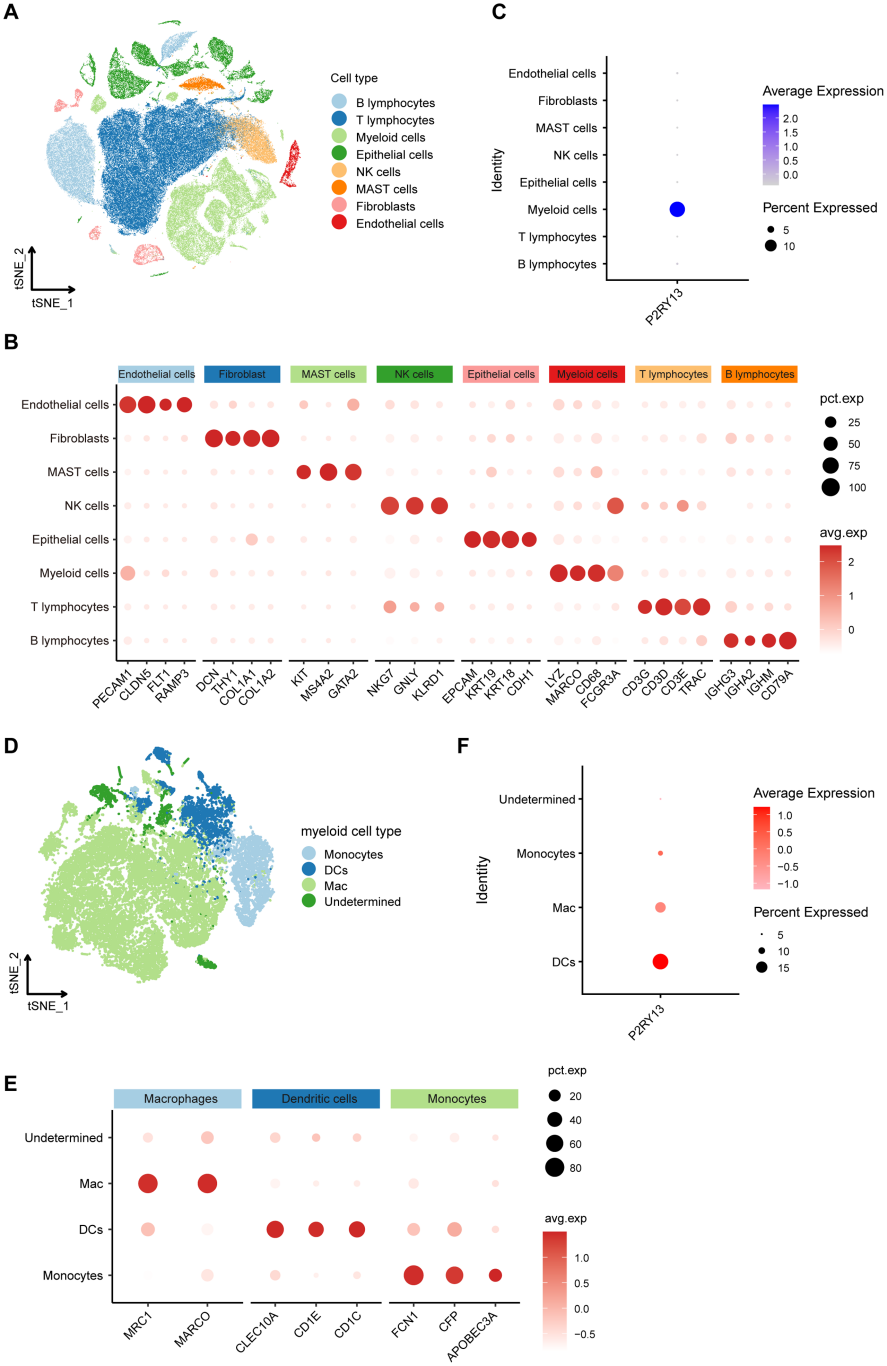
Single-cell profiling identifies dendritic cells as the primary source of P2RY13 expression (GSE131907). **(A)** t-SNE visualization of major immune cell populations identified in the GSE131907 dataset. **(B)** Expression of canonical marker genes for each immune cell type. **(C)** Expression pattern of P2RY13 across immune cell subtypes, indicating predominant enrichment in myeloid cells. **(D)** Re-clustering of myeloid cells into subsets: DCs, Mac, monocytes, and undetermined cells. **(E)** Expression of canonical marker genes for DCs, Mac, and monocytes. **(F)** P2RY13 expression across myeloid cell subtypes, revealing predominant expression in DCs.

A total of 57,910 T cells, 16,999 B cells, 1996 endothelial cells, 17,555 epithelial cells, 3,499 fibroblasts, 2,888 mast cells, 28,083 myeloid cells, and 8,411 NK cells were identified (Figure 5A). Marker genes for each major cell population were shown in Figure 5B. P2RY13 expression was found to be predominantly enriched in myeloid cells (Figure 5C). We subsequently subset and re-clustered the myeloid compartment, annotating 3,296 DCs, 19,682 Mac, 3,489 monocytes, and 1,616 undetermined cells based on established marker genes (Figures 5D,E). Further analysis revealed that P2RY13 was primarily expressed by DCs (Figure 5F). These results suggest that P2RY13 may be functionally implicated in antigen presentation processes mediated by DCs.

### 3.5 P2RY13 was associated with antigen presentation and activation of T cells

**Figure 6:**
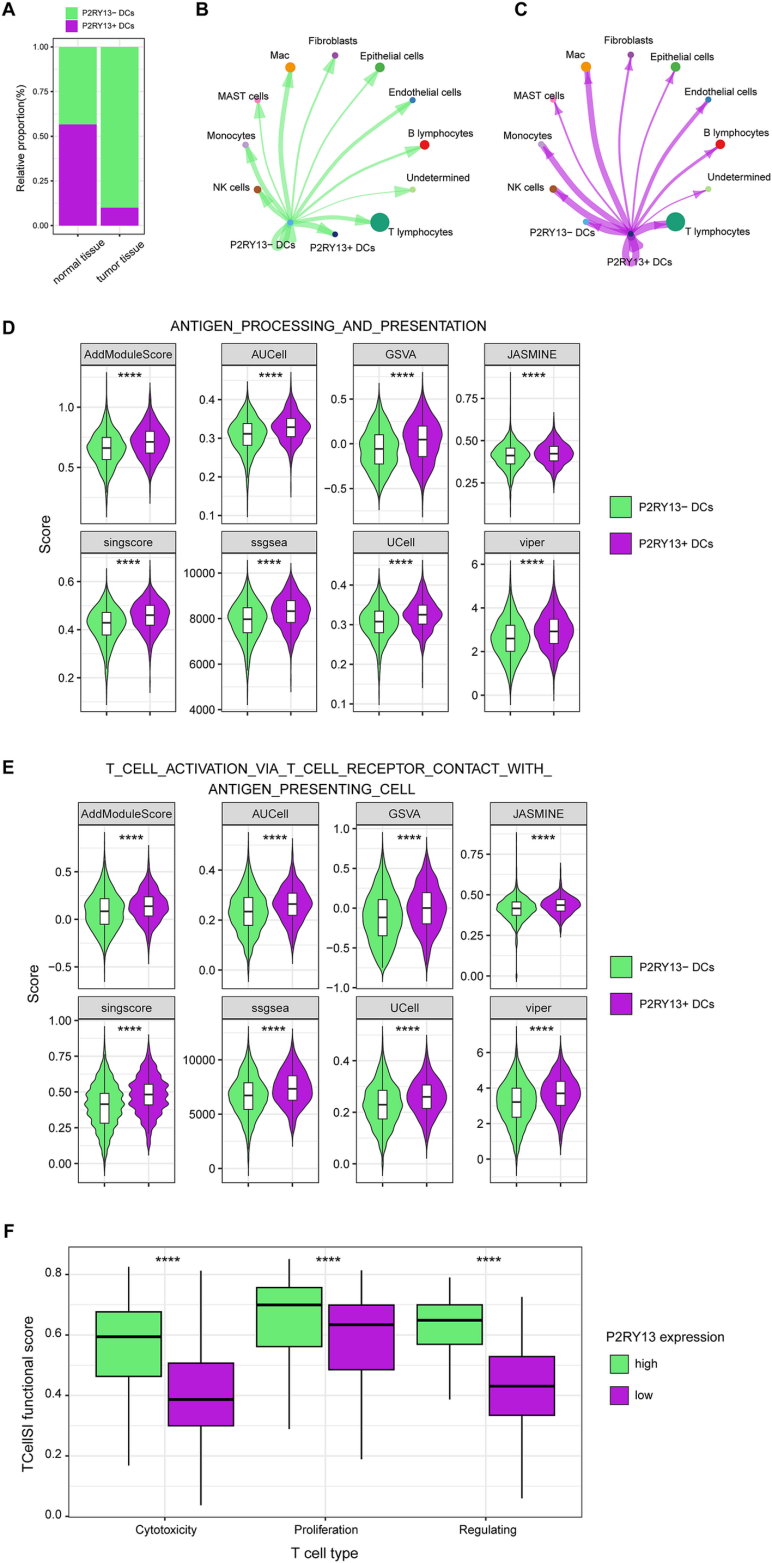
P2YR13(+) DCs correlated with enhanced T cell activation. **(A)** The proportion of P2RY13(+) DCs was significantly lower in tumor samples compared to normal tissues. **(B,C)** CellChat analysis depicting the outgoing interaction strength of **(B)** P2RY13(−) DCs and **(C)** P2RY13(+) DCs toward other immune cells. **(D,E)** Comparison of **(D)** antigen processing and presentation pathway activity and **(E)** T cell activation pathway activity between P2RY13(+) and P2RY13(−) DCs by eight single-cell signature scoring methods. **(F)** TCellSI-derived functional scores demonstrated higher T cell activation in the P2RY13 high-expression group compared to the low-expression group. ^****^p < 0.0001.

Based on P2RY13 expression levels, DCs were classified into 647 P2RY13(+) DCs and 2,649 P2RY13(−) DCs. P2RY13(+) DCs were significantly more abundant in normal tissues compared to tumor samples (56.6% vs. 10.0%; *p* < 0.05; Figure 6A), consistent with bulk-seq transcriptome findings (Figure 1A). CellChat analysis revealed enhanced interactions between P2RY13(+) DCs and T cells compared to P2RY13(−) DCs (Figures 6B,C). Using eight single-cell scoring methods, we consistently observed elevated activity of antigen processing and presentation pathways and T cell activation pathways in P2RY13(+) DCs (Figures 6D,E). In TCGA-LUAD bulk-seq dataset, TCellSI functional scores demonstrated elevated activity levels in cytotoxic T cells, proliferative T cells, and regulatory T cells in patients with P2RY13 high expression compared to those with low expression (*p* < 0.001, Figure 6F). These results suggest that P2RY13 expression may enhance antigen presentation and T cell activation functions in DCs.

## 4 Discussion

Our study provided compelling multi-omics evidence that P2RY13 was not only a prognostic biomarker but also a potential immune modulator within the LUAD microenvironment. The consistent downregulation of P2RY13 in tumor tissues and its strong association with unfavorable survival outcomes highlight its clinical relevance. More importantly, through single-cell resolution analysis, we identified DCs as the key cellular compartment expressing P2RY13, with P2RY13(+) DCs exhibiting enhanced antigen presentation machinery and increased interactions with lymphocytes, especially T cells. This cellular specificity represents a significant advancement over previous study that merely reported bulk tissue expression patterns of P2RY13 without cellular context (11).

The implications of a loss of P2RY13(+) DCs for anti-tumor immunity are substantial. DCs play a central role in initiating and modulating anti-tumor immune responses in NSCLC by presenting tumor antigens and providing co-stimulatory signals to T cells (13–15). However, the function of DCs is frequently suppressed within the LUAD TME, contributing to immune evasion (16, 17). The preferential localization of P2RY13(+) DCs in normal tissues in the present study further supports the concept that loss of this DCs subpopulation may contribute to immunosuppression in LUAD. The observed reduction of P2RY13(+) DCs within LUAD tumors, suggests that this specific DCs subset is either excluded, suppressed, or fails to develop within the TME. Our findings that P2RY13 expression correlates with an immunostimulatory DCs phenotype and enhanced T cell activation align with this paradigm, suggesting that modulating P2RY13 signaling could be a viable strategy to reinvigorate the anti-tumor immune response. Emerging computational pharmacology frameworks, such as COIMMR, are now capable of quantifying the contribution of drugs, including natural products, to anti-tumor efficacy through the specific modulation of the immune microenvironment (18). From a pharmacological standpoint, P2RY13 represents a promising therapeutic target as a G protein-coupled receptor (GPCR), a class with high druggability. This potential is further supported by pharmacotranscriptomic approaches, exemplified by the Integrated Traditional Chinese Medicine (ITCM) platform, which has successfully identified immunomodulatory natural products targeting similar receptors, thereby providing a validated strategy for developing P2RY13 modulators (19).

A key question emerging from our findings is the mechanism of P2RY13 downregulation in DCs within the TME. Although not directly tested here, we propose several plausible explanations based on known TME biology. First, the metabolically adverse TME, characterized by hypoxia and lactate accumulation, can broadly reshape transcriptional landscapes in immune cells (20). Purinergic receptors are modulated by extracellular metabolites, and their dysregulation may arise from such perturbations (21). Additionally, epigenetic mechanisms, including promoter DNA methylation of P2RY13 in tumor-infiltrating DCs, may contribute to its transcriptional silencing. Further studies are needed to clarify the dominant pathway underlying P2RY13 suppression.

Although prior study has reported the downregulation of P2RY13 in LUAD and its correlation with immune infiltration, the specific cell types mediating these effects remained unclear (11). Our study provides the first evidence that P2RY13 is preferentially expressed in DCs and is associated with their immunostimulatory phenotype. This aligns with clinical data showing that high P2RY13 expression correlates with improved prognosis, likely due to more effective anti-tumor immunity.

While our study focused on LUAD, the role of purinergic signaling in immune regulation suggests that P2RY13 might have broader implications in cancer immunity. The P2Y receptor family has been implicated in various immune processes, including cytokine secretion (22), cell migration (23), and activation of DNA repair processes (24). However, the exact mechanisms through which P2RY13 regulates DCs function remain to be elucidated. Future studies should investigate whether P2RY13 activation enhances cross-presentation capacity, promotes DCs maturation, or facilitates migration to lymph nodes.

Several limitations warrant consideration when interpreting our results. The retrospective nature of the analysis and reliance on computational methods for immune cell quantification necessitate experimental validation. The use of public datasets, while providing substantial statistical power, may introduce cohort-specific biases. Additionally, the functional characterization of P2RY13in DCs remains inferential based on transcriptional signatures. Future studies employing genetic manipulation of P2RY13 in DCs, followed by functional assays and animal models, will be crucial for validating the role of P2RY13 in DC-dependent lymphocyte activation and its therapeutic potential.

## 5 Conclusion

P2RY13 was significantly downregulated in LUAD and served as an independent prognostic factor. We identified DCs as the primary source of P2RY13 within the TME and revealed its association with enhanced antigen presentation and lymphocyte activation.

## Data Availability

The datasets (GSE68465 and GSE31210) analyzed in the present study were derived from publicly accessible databases (TCGA: https://portal.gdc.cancer.gov/; GEO: https://www.ncbi.nlm.nih.gov/geo/).
